# Dysfunction of the circadian transcriptional factor CLOCK in mice resists chemical carcinogen-induced tumorigenesis

**DOI:** 10.1038/s41598-017-10599-1

**Published:** 2017-08-30

**Authors:** Ken-ichi Hashikawa, Chiharu Katamune, Naoki Kusunose, Naoya Matsunaga, Satoru Koyanagi, Shigehiro Ohdo

**Affiliations:** 1 Department of Pharmaceutics, Faculty of Pharmaceutical Sciences, Kyushu University, 3-1-1 Maidashi Higashi-ku, Fukuoka, 812-8582 Japan; 2 Department of Glocal Healthcare Science, Faculty of Pharmaceutical Sciences, Kyushu University, 3-1-1 Maidashi Higashi-ku, Fukuoka, 812-8582 Japan

## Abstract

The chronic disruption of circadian rhythms has been implicated in the risk of cancer development in humans and laboratory animals. The gene product CLOCK is a core molecular component of the circadian oscillator, so that mice with a mutated *Clock* gene (*Clk/Clk*) exhibit abnormal rhythms in various physiological processes. However, we demonstrated here that *Clk/Clk* mice resisted chemical carcinogen-induced tumorigenesis by suppressing epidermal growth factor (EGF) receptor-mediated proliferation signals. The repetitive application of 7,12-dimethylbenz[α]anthracene (DMBA) to skin on the back resulted in the significant development of tumors in wild-type mice, whereas chemically-induced tumorigenesis was alleviated in *Clk/Clk* mice. Although the degree of DMBA-induced DNA damage was not significantly different between wild-type and *Clk/Clk* mice, EGF receptor-mediated Ras activation was not detected in DMBA-treated *Clk/Clk* mice. Genetic and biochemical experiments revealed that the suppression of EGF receptor-mediated signal transduction in DMBA-treated *Clk/Clk* mice was associated with the expression of the cellular senescence factor p16INK4a. These results suggest an uncovered role for CLOCK in the development of chemical carcinogen-induced primary tumors and offers new preventive strategies.

## Introduction

In mammals, circadian rhythms in physiological functions are generated by a molecular oscillator driven by a transcriptional-translational feedback loop consisting of negative and positive regulators^1–3^. The gene products of *Clock* and *Bmal1* (also known as *Arntl*) form a heterodimer that acts as a positive transcription factor to activate the transcription of the *Period* (*Per*) and *Cryptochrome* (*Cry*) genes. Once the PER and CRY proteins have reached a critical concentration, they act as negative transcription factors to attenuate CLOCK/BMAL1-mediated transactivation. The alternating activation and repression of the CLOCK/BMAL1-driven positive loop and PER/CRY-controlled negative loop result in a circadian oscillation in the molecular clock^4, 5^ and also allow organisms to synchronize their physiological and behavioral functions to anticipatory changes in their environment^6–8^. Therefore, disruption of the circadian clock has detrimental effects on health and increases the risk of cancer development^9–11^.

In fact, human night shift workers are at an increased risk of developing breast, prostate, colon, and endometrial cancers as well as non-Hodgkin lymphoma^12–16^. These epidemiological findings are supported by animal studies in which repetitive changes in the light-dark cycle were found to facilitate the growth of implanted tumors^17^. Although the genetic ablation of negative or positive components of the circadian clock leads to disrupted rhythms in physiological functions, we previously demonstrated the different contributions of each component of the circadian clock to oncogene-induced cellular transformation^18^. Mouse embryonic fibroblasts prepared from animals deficient in the negative circadian clock regulators, *Per2* or *Cry1/2*, were susceptible to transformation induced by the co-expression of H-Ras^V12^ and SV40 large T antigen (SV40LT). Consistent with these findings, mice with a mutated *Per2* gene (*Per2* ^m/m^) were predisposed to spontaneous as well as radiation-induced tumor development^19–21^. On the other hand, cells prepared from mice deficient in the positive circadian clock regulators, *Clock* or *Bmal1*, showed resistance to oncogene-induced transformation. However, the tumorigenic phenotype of mice deficient in positive circadian clock regulators currently remains unknown.

Mice with a mutated *Clock* gene exhibit abnormal rhythms in physiology and behavior^22–24^. We herein demonstrated that *Clk*/*Clk* mice resisted chemical carcinogen-induced tumorigenesis by suppressing EGF receptor-mediated proliferation signals. The application of DMBA to the back skin of *Clk/Clk* mice induced the expression of the cellular senescence factor p16INK4a, which prevents EGF receptor-mediated Ras activation. Collectively, the results of the present study suggest a role for CLOCK in the development of chemically-induced primary tumors and will contribute to new preventive strategies.

## Results

### Resistance of *Clk/Clk* mice to the DMBA-induced development of skin tumors

**Figure 1:**
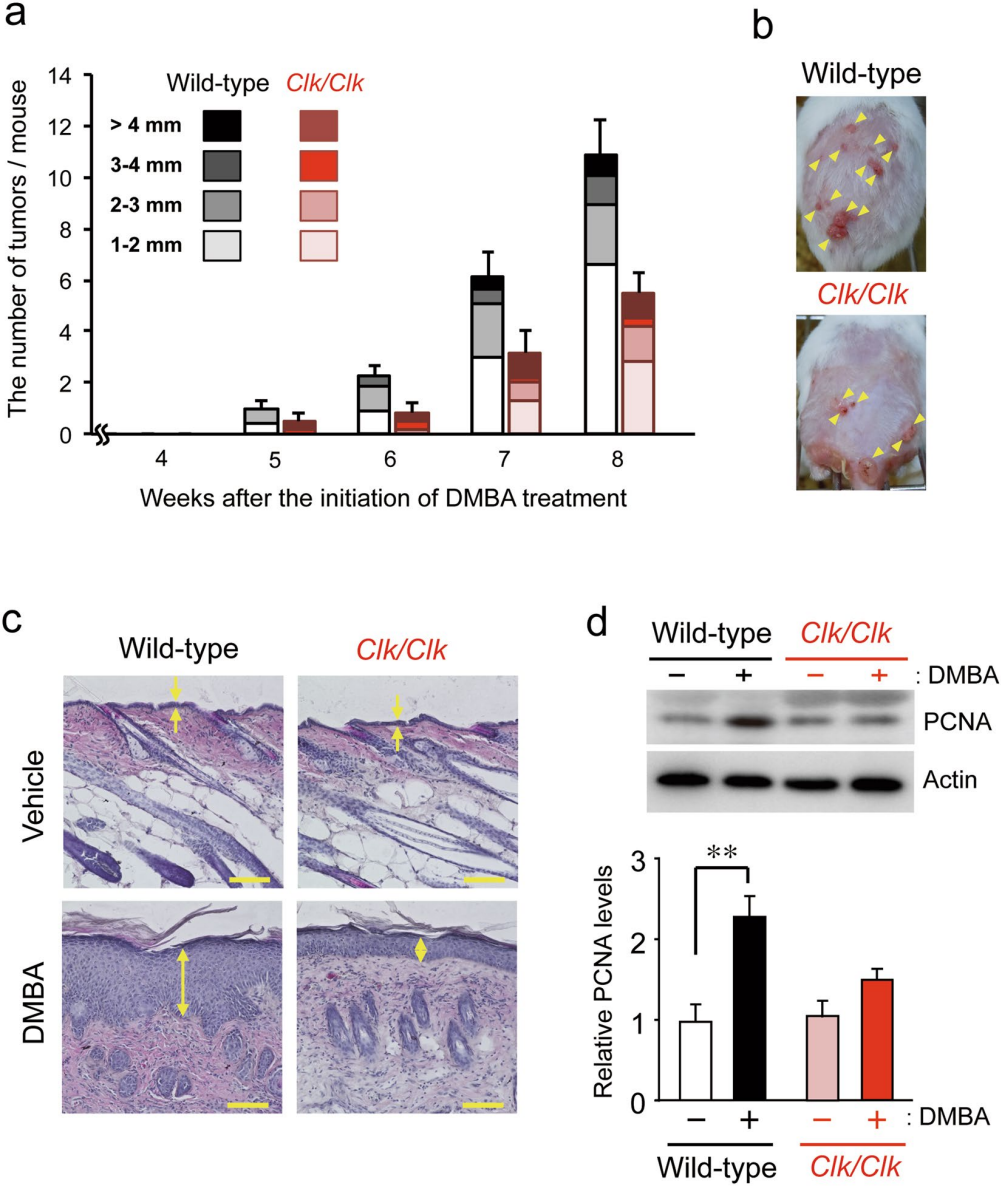
Comparison of DMBA-induced skin tumorigenesis between wild-type and *Clk*/*Clk* mice. Animals were treated with 100 μg DMBA twice a week. (**a**) Time course of DMBA-induced skin tumor formation in wild-type and *Clk/Clk* mice. Each column indicates the average number of tumors and their size distribution obtained from 6–12 mice per each group (means ± s.e.m.). Tumors with a diameter of more than 1 mm were counted every week. DMBA-induced tumorigenesis was significantly alleviated in *Clk/Clk* mice (*F*_*1*,*16*_ = 6.302, *P* = 0.023; two-way repeated measures ANOVA). (**b**) Representative photographs of skin tumor formation in wild-type and *Clk/Clk* mice 8 weeks after initiation of the DMBA treatment. The yellow arrows indicate the formation of tumors. (**c**) Histological analysis of the dorsal skin of wild-type and *Clk/Clk* mice after the DMBA treatment. Mice were treated with DMBA or vehicle (200 μL acetone) for 2 weeks. Skin samples were stained with hematoxylin and eosin. Yellow arrows indicate the epidermis. Scale bar, 100 μm. Histological data were confirmed in more than three mice in each group. (**d**) The protein levels of proliferating cell nuclear antigen (PCNA) in the skin of wild-type and *Clk/Clk* mice 2 weeks after initiation of the DMBA treatment. Full-size images are presented in Supplementary Fig. 2. Values show the means ± s.e.m. (*n* = 3). **P < 0.01 significant difference between two groups (*F*_3,8_ = 9.664, *P* = 0.005; ANOVA with Tukey-Kramer’s post-hoc test).

To investigate the role of the *Clock* gene in tumorigenesis, wild-type and *Clk/Clk* mice were subjected to a conventional chemically-induced skin tumor protocol with the biweekly application of DMBA for 8 weeks. This protocol was employed because DMBA is known to induce the development of skin tumors^25–27^. In wild-type and *Clk/Clk* mice, tumors with a diameter of more than 1 mm appeared 5 weeks after the initiation of the DMBA treatment (Fig. 1a and b). The numbers and sizes of tumors gradually increased during the duration of the experiment; however, chemical carcinogen-induced tumorigenesis was significantly alleviated in *Clk/Clk* mice (*F*_*1*,*16*_ = 6.302, *P* <0.05; Fig. 1a). The results of the histological analysis also revealed that obvious epidermal hyperplasia was induced in wild-type mice 2 weeks after the initiation of the DMBA treatment, during which tumors had not yet been observed on the skin (Fig. 1c). Since the expression of proliferating cell nuclear antigen (PCNA) was significantly increased in the skin of DMBA-treated wild type mice (*P* < 0.01; Fig. 1d), application of the chemical carcinogen appears to induce the transition of skin cells into proliferative state. In contrast to wild-type mice, DMBA-induced hyperplasia and PCNA expression were attenuated in *Clk/Clk* mice. These results suggest that *Clk/Clk* mice resist chemical carcinogen-induced tumorigenesis.

### DMBA-induced DNA damage to the skin of wild-type and *Clk/Clk* mice

**Figure 2:**
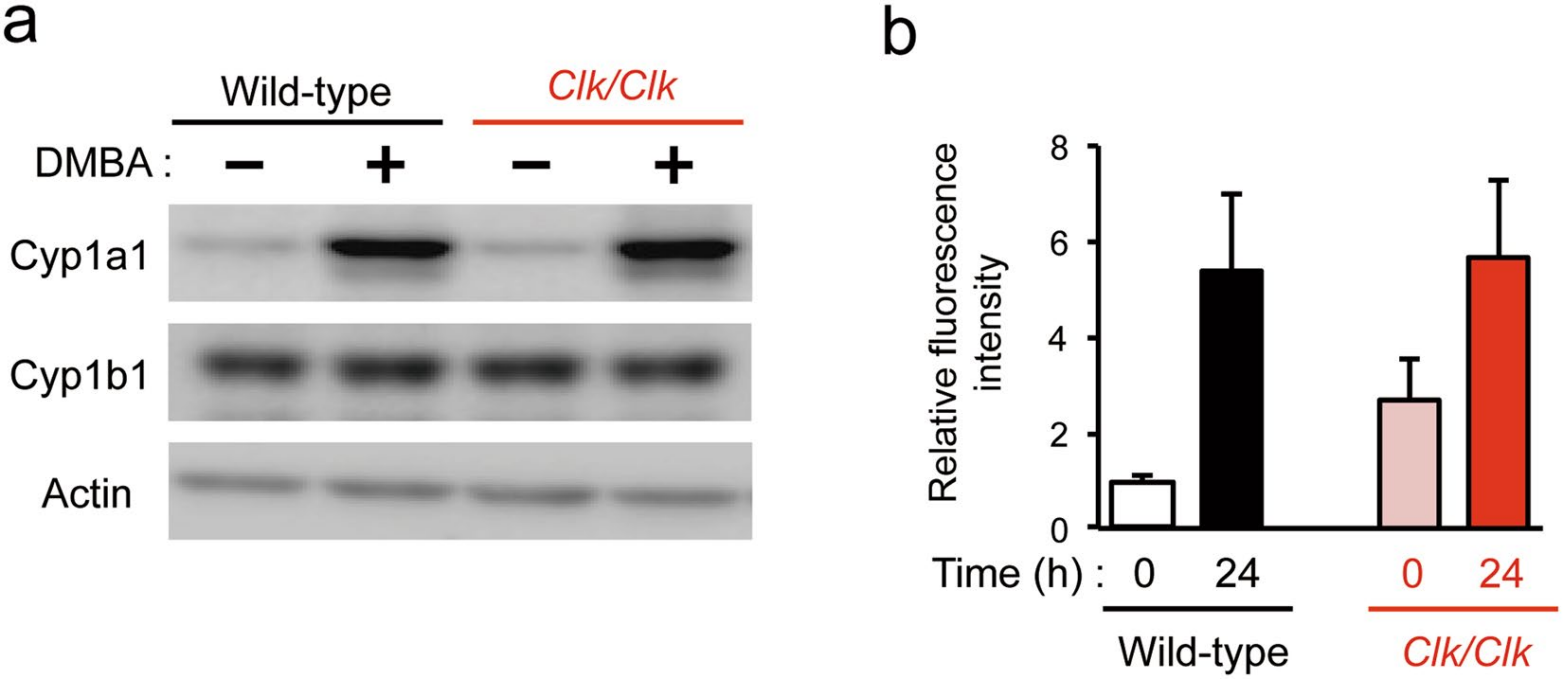
Induction of Cyp proteins and formation of DNA adducts in the skin of wild-type and *Clk/Clk* mice after the DMBA treatment. (**a**) Protein levels of Cyp1a1 and Cyp1b1 in the skin of wild-type and *Clk/Clk* mice after the DMBA treatment. Skin homogenates were prepared 12 hours after the treatment with 100 μg DMBA. Plus and minus indicate the treatment with DMBA and vehicle (200 μL acetone), respectively. Full-size images are presented in Supplementary Fig. 3. Western blotting data were confirmed in more than three mice in each group. (**b**) Amounts of DMBA-DNA adducts in the skin of wild-type and *Clk/Clk* mice. DMBA was applied twice at 8-hour intervals. DNA was extracted from the skin of mice at 0 or 24 hours after the second DMBA treatment. The fluorescence intensity derived from DMBA-DNA adducts was measured, and intensity was normalized by DNA contents in samples (Means ± s.e.m.; *n* = 3–4).

DMBA is converted to its bioactive form after being metabolized by Cyp1a1 and Cyp1b1^28, 29^ and then causes DNA damage by forming adducts with the nucleobase^30, 31^. To elucidate the mechanisms responsible for differences in tumorigenicity between wild-type and *Clk*/*Clk* mice, we assessed the protein levels of these enzymes in the skin of wild-type and *Clk*/*Clk* mice after the DMBA treatment. Consistent with previous findings^32^, the protein levels of Cyp1a1, but not Cyp1b1 in the skin of wild-type mice were increased 12 hours after a single dose of DMBA (Fig. 2a). The similar induction of Cyp1a1 expression was also detected in *Clk/Clk* mice, but with no significant differences in the DMBA-induced expression of metabolic enzymes between wild-type and *Clk/Clk* mice.

We also evaluated DNA damage in the skin of mice after the DMBA treatment by measuring the intensity of fluorescence derived from DMBA-DNA adducts^33^. Since a single dose of DMBA was insufficient to detect fluorescence intensity, the amount of DMBA-DNA adducts was measured after the application of DMBA twice to mouse skin at 8-hour intervals. In wild-type and *Clk*/*Clk* mice, the intensity of fluorescence derived from DMBA-DNA adducts was detected 24 hours after the second application of DMBA, while no significant differences were observed between the genotypes (Fig. 2b). These results suggest that the degree of DMBA-induced DNA damage in *Clk/Clk* mice was similar to that observed in wild-type mice.

### Influence of the *Clock* gene mutation on cell proliferation signals after the DMBA treatment

**Figure 3:**
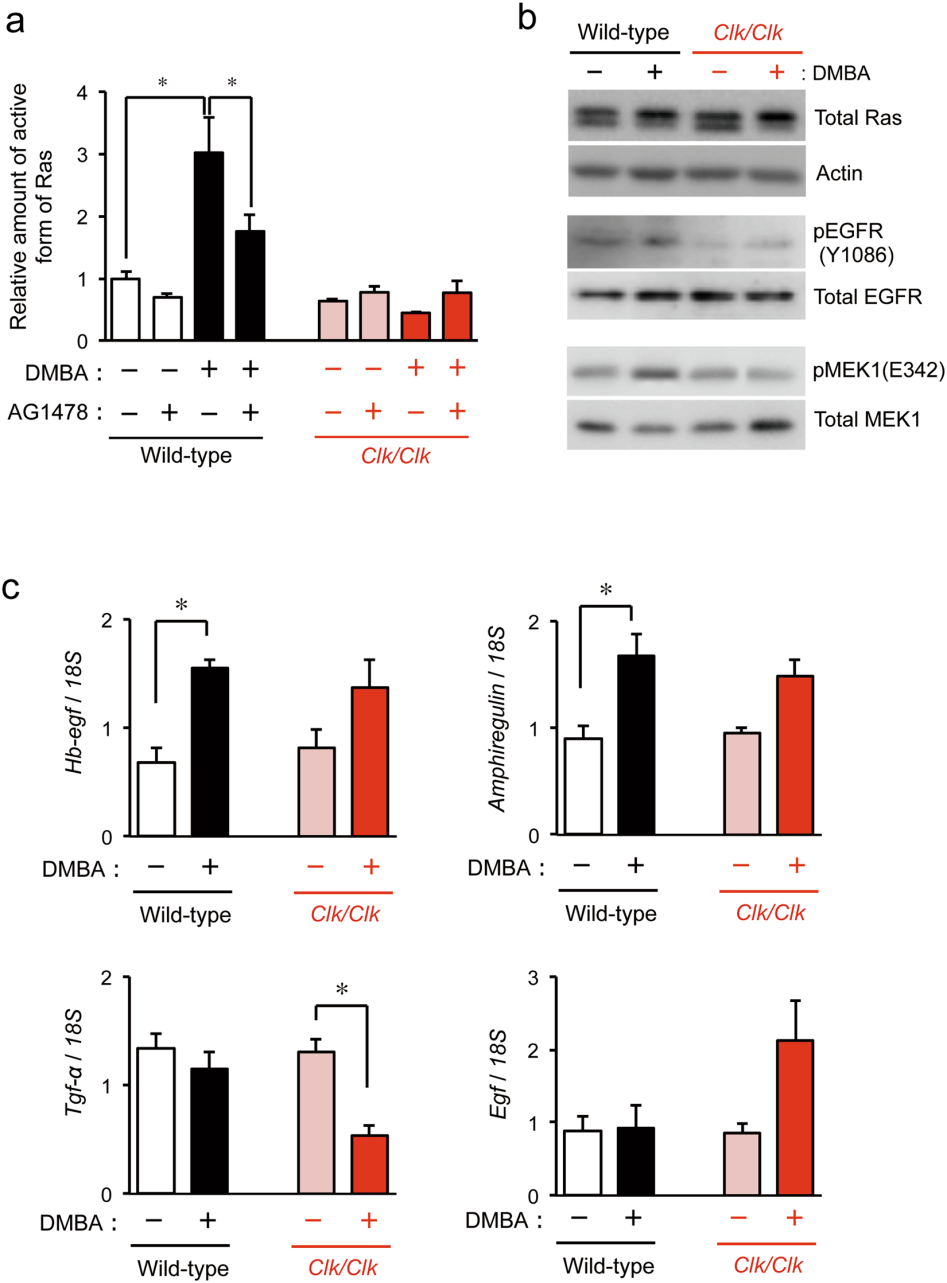
Biochemical analysis of cell proliferation signaling in the skin of wild-type and *Clk/Clk* mice after the DMBA treatment. DMBA (100 μg) or vehicle (200 μL acetone) was applied biweekly to the back skin of mice for 2 weeks. Plus and minus indicate the treatment with DMBA and vehicle, respectively. (**a**) Amount of the active form of Ras in the skin of wild-type and *Clk/Clk* mice 2 weeks after initiation of the DMBA treatment. AG1478 (100 μg) was applied to the dorsal skin of mice 3 hours before sampling. Values of absorbance at 450 nm were obtained and normalized by total protein levels (means ± s.e.m.; *n* = 5). *P < 0.05 significant difference between the two groups (*F*_*7*,*23*_ = 11.945, *P* < 0.001; ANOVA with Tukey-Kramer’s post-hoc test). (**b**) The protein levels of all (active and inactive) forms of Ras, phosphorylated EGF receptor (pEGFR), and phosphorylated MEK1 (pMEK1) in the skin of wild-type and *Clk/Clk* mice 2 weeks after initiation of the DMBA treatment. Full-size images are presented in Supplementary Fig. 4. Western blotting data were confirmed in more than three mice in each group. (**c**) mRNA levels of endogenous ligands for the EGF receptor in the skin of wild-type and *Clk/Clk* mice after initiation of the DMBA treatment (Means ± s.e.m.; *n* = 5). *P < 0.05 significant difference between the two groups (*F*_*3*,*16*_ = 5.986, *P* = 0.006 for *Hb-egf*, *F*_*3*,*16*_ = 7.257, *P* = 0.003 for *Amphiregulin*, *F*_*3*,*16*_ = 8.365, *P* = 0.001 for *Tgf-α*; ANOVA with Tukey-Kramer’s post-hoc test).

Ras, a small GTP-binding protein, is an important component of the signal transduction pathway used by growth factors to initiate cell growth and differentiation^34^. Although the aberrant activation of Ras by non-hydrolyzable GTP-binding mutations is a major cause of tumorigenesis^35, 36^, this GTP-binding protein also contributes to the wound healing process^37^. The repetitive application (twice a week) of DMBA to the back skin of wild-type mice for two weeks caused elevations in the levels of the active form of Ras (*P* < 0.05; Fig. 3a). Since the total amount of Ras was not markedly affected by the DMBA treatment (Fig. 3b, top panel), elevated levels of the active form of Ras in DMBA-treated wild-type mice were unlikely to be associated with changes in its protein abundance. Furthermore, the activation of Ras in DMBA-treated wild-type mice was significantly suppressed by the selective EGF receptor inhibitor AG1478 (*P* < 0.05, Fig. 3a), suggesting that Ras activation is due to the stimulation of EGF receptors with its cognate ligands. In fact, we also found the enhancement of phosphorylation of EGF receptor in the skin of DMBA-treated wild-type mice (Fig. 3b, middle panel). In contrast to wild-type mice, the same treatment with DMBA did not induce the activation of Ras in the skin of *Clk/Clk* mice (Fig. 3a). Since phosphorylation of EGF receptor in *Clk/Clk* mice was also not induced by DMBA treatment (Fig. 3b, middle panel), the failure to activate Ras in DMBA-treated *Clk/Clk* mice appeared to be associated with inactivation of EGF receptor. Activated Ras enhances the protein kinase activity of Raf, which subsequently phosphorylates and activates MEK, resulting in the propagation of the signal to downstream effectors, such as MAPK^38^. Consistent with Ras activation, the phosphorylation state of MEK was also elevated in DMBA-treated wild-type mice (Fig. 3b, bottom panel), but the DMBA-induced MEK phosphorylation was not induced in *Clk/Clk* mice.

After ligand binding, the EGF receptor forms a homodimeric complex and rapidly activates Ras via several adaptor proteins^39, 40^. EGF, heparin-binding EGF-like growth factor (HB-EGF), transforming growth factor-α (TGF-α), and amphiregulin have been identified as endogenous ligands of EGF receptors^41–44^. After the biweekly treatment with DMBA for 2 weeks, the mRNA levels of *Hb-egf* and *Amphiregulin* increased in wild-type and *Clk/Clk* mice (Fig. 3c). The expression of *Tgf-α* mRNA in wild-type mice was not significantly affected by the DMBA treatment, whereas these mRNA levels were significantly decreased in DMBA-treated *Clk/Clk* mice (*P* < 0.05). In contrast to *Tgf-α*, the mRNA levels of *Egf* slightly increased in DMBA-treated *Clk/Clk* mice (*P* = 0.074). These results suggest that some types of ligands for the EGF receptor are involved in the activation of Ras in wild-type mice during chemical carcinogen-induced tumorigenesis. However, the expression profiles of ligands for the EGF receptor were unable to explain failed Ras activation in DMBA-treated *Clk/Clk* mice.

### Induction of apoptotic cells and cellular senescence in DMBA-treated *Clk*/*Clk* mice

**Figure 4:**
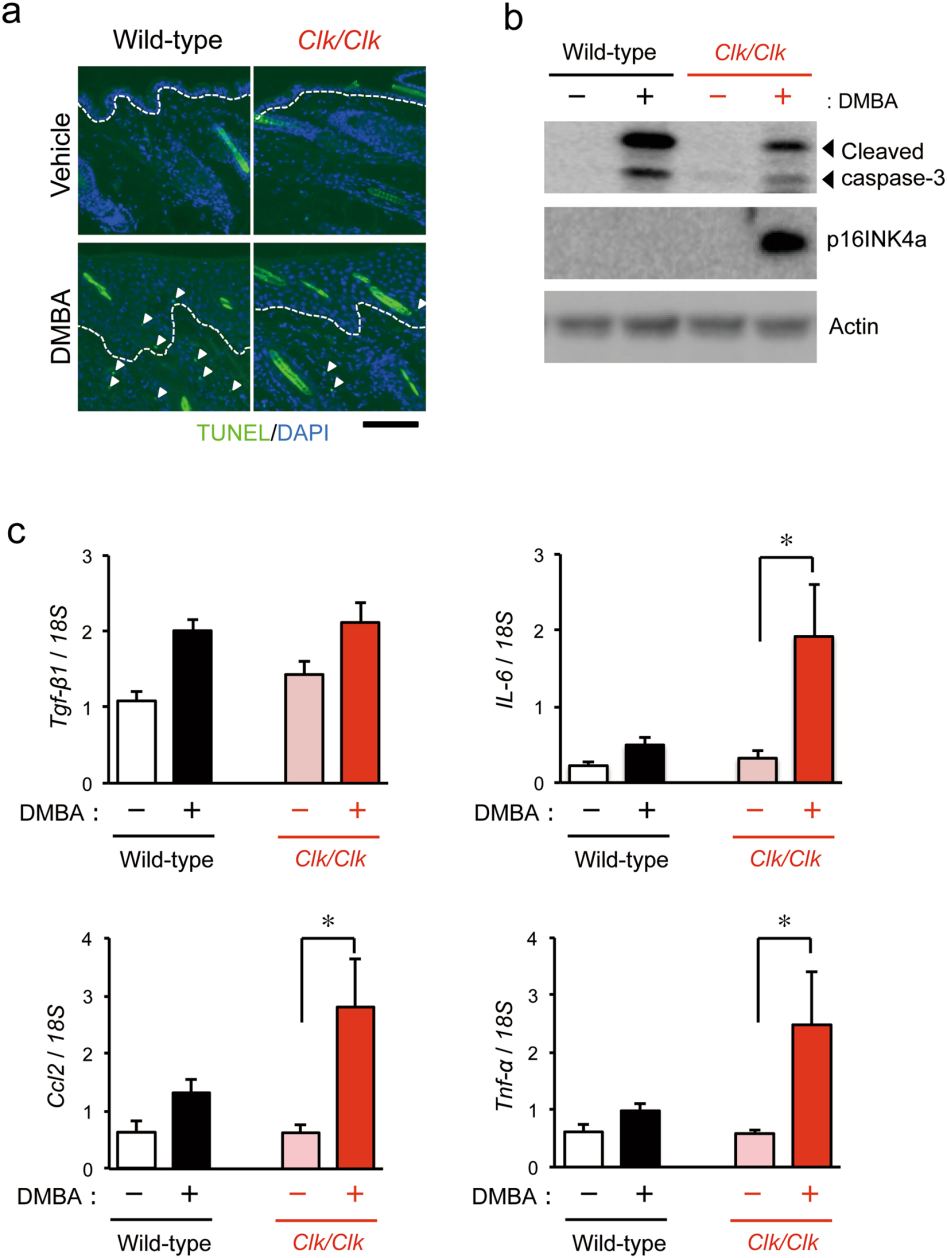
DMBA-induced apoptotic cell death and cellular senescence in the skin of wild-type and *Clk*/*Clk* mice. Animals were treated with 100 μg DMBA or vehicle (200 μL acetone) twice a week. (**a**) Immunofluorescence staining of apoptotic cells in the skin of wild-type and *Clk/Clk* mice 2 weeks after initiation of the DMBA treatment. Apoptotic cells were detected by the TUNEL assay (Green). DAPI was used to stain nuclei (Blue). White arrows indicate TUNEL-positive cells. Dashed lines denote the epidermis-dermis border. Scale bar, 100 μm. (**b**) The expression of cleaved caspase-3 and p16INK4a proteins in the skin of wild-type and *Clk/Clk* mice 2 weeks after initiation of the DMBA treatment. Plus and minus indicate the treatment with DMBA and vehicle (200 μL acetone), respectively. Full-size images are presented in Supplementary Fig. 5. Data shown in panel a and b were confirmed in more than three mice in each group. (**c**) mRNA levels of inflammatory cytokines in the skin of wild-type and *Clk/Clk* mice 2 weeks after initiation of the DMBA treatment. (Means ± s.e.m.; *n* = 5). *P < 0.05 significant difference between the two groups (*F*_*3*,*14*_ = 6.412, *P* = 0.006 for *IL-6*, *F*_*3*,*16*_ = 5.211, *P* = 0.011 for *Ccl2*, *F*_*3*,*16*_ = 3.682, *P* = 0.034 for *Tnf-α*; ANOVA with Tukey-Kramer’s post-hoc test). Plus and minus indicate the treatment with DMBA and vehicle (200 μL acetone), respectively.

During oncogenic transformation, cells acquire genetic mutations that override the normal cell-cycle mechanism, resulting in abnormal proliferation. The failure of cells to override this mechanism often causes reversible cell-cycle arrest, apoptotic cell death, or cellular senescence^45^. Therefore, we also investigated cell death events and senescence in the skin of mice after the DMBA treatment. The results of the histochemical analysis using the terminal deoxynucleotidyltransferase (TdT)-mediated dUTP-digoxigenin nick-end labeling (TUNEL) assay revealed that the number of apoptotic cells in DMBA-treated *Clk/Clk* mice was less than that in wild-type mice (Fig. 4a). Consistent with this result, the protein levels of cleaved caspase-3 were also lower in *Clk/Clk* mice than in wild-type mice (Fig. 4b).

The tumor suppressor gene *Cdkn2a* acts as a canonical inducer of cellular senescence^46^. *Cdkn2a* generates the different transcript variants, p16Ink4a and p19Arf (also known as p14ARF in humans), by using different first exons and alternate polyadenylation sites^47^. The p16Ink4a variants encode structurally related protein isoforms that inhibit CDK4 kinase^48^. The CDK4 inhibitor prevents the phosphorylation of pRB by disrupting the activity of the CDK4-cyclin D complex. On the other hand, p19ARF stabilizes p53 by sequestering MDM2^49^. The protein levels of p16INK4a in the skin of *Clk/Clk* mice were significantly increased by the DMBA treatment, whereas no obvious band derived from p16INK4a was detected in wild-type mice (Fig. 4b). We also attempted to assess the protein levels of p19ARF in DMBA-treated mice; however, this protein was not detected in the skin of mice.

Since senescent cells show the senescence-associated secretory phenotype (SASP) reflecting the production of inflammatory cytokines and chemokines^50, 51^, we investigated the expression of typical inflammatory cytokines and chemokines in the skin of DMBA-treated mice. In wild-type and *Clk/Clk* mice, the mRNA levels of *Tgf-β1* increased after the DMBA treatment, while expression levels were not significantly different between the genotypes (Fig. 4c). On the other hand, the expression of *IL-6*, *Ccl2*, and *Tnf-α* was significantly increased only in DMBA-treated *Clk/Clk* mice (*P* < 0.05, respectively), suggesting that the treatment of *Clk/Clk* mice with DMBA induces cellular senescence rather than neoplastic transformation.

### The expression of p16INK4a prevents EGF receptor-mediated Ras activation

**Figure 5:**
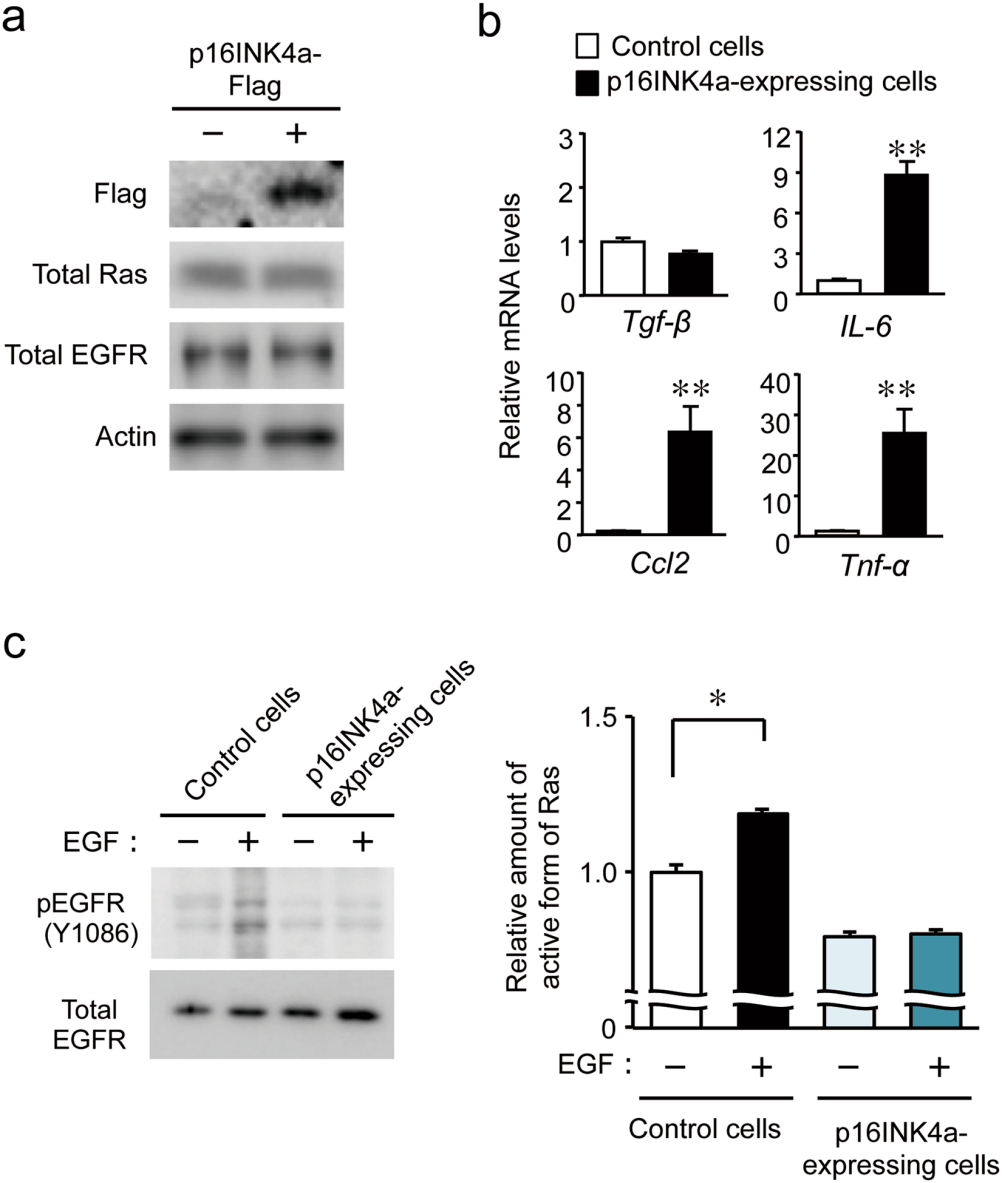
Effects of p16INK4a on EGF receptor-mediated Ras activation in human keratinocytes. HaCaT cells were infected with retroviral vectors expressing Flag-tagged p16INK4a. (**a**) Protein levels of Flag-tagged p16INK4a, total Ras, and EGF receptor in HaCaT cells. Plus and minus indicate the infection with p16INK4a-Flag expressing vectors. Full-size images are presented in Supplementary Fig. 6. Data shown were confirmed in more than three independent experiments. (**b**) mRNA levels of inflammatory cytokines in cells infected or not infected with the p16INK4a-Flag expressing vectors. (Means ± s.e.m.; *n* = 6). **P < 0.01 significant difference control cells (*t*_10_ = −8.393, *P* < 0.001 for *IL-6*, *t*_10_ = −4.161, *P* = 0.002 for *Ccl2*, *t*_10_ = −3.975, *P* = 0.034 for *Tnf-α*; unpaired *t*-test, two-sided). (**c**) Protein levels of phosphorylated EGFR (right) and the amount of the active form of Ras (left) in cells infected or not infected with the p16INK4a-Flag expressing vectors. For right panel, western blotting data were confirmed in three mice in each group. Full-size images are presented in Supplementary Fig. 7. For left panel, cells were incubated in serum-starved media for 24 hours and then stimulated with 200 ng/ml of EGF for 1 minute. Plus and minus indicate the introduction of the p16INK4a-Flag vector and the EGF treatment, respectively. Values of absorbance at 450 nm were obtained and normalized by total protein levels (Means ± s.e.m.; *n* = 4). *P < 0.05 significant difference between the two groups (*F*_*3*,*12*_ = 29.245, *P* < 0.001; ANOVA with Tukey-Kramer’s post-hoc test).

In contrast to reversible cell-cycle arrest, cellular senescence is defined by the irreversible loss of proliferative potential^52, 53^. In the final set of experiments, we used cultured human keratinocyte HaCaT cells to investigate whether elevation of p16INK4a protein levels prevents EGF receptor-mediated Ras activation. The infection of HaCaT cells with retroviral vectors expressing flag-tagged human p16INK4a had a negligible effect on the protein levels of Ras and the EGF receptor (Fig. 5a). The mRNA levels of *IL-6*, *Ccl2*, and *Tnf-α* were significantly increased in p16INK4a-expressing cells (P < 0.01, respectively; Fig. 5b). The treatment of control HaCaT cells with EGF significantly induced the phosphorylation of EGF receptor as well as activation of Ras (*P* < 0.05; Fig. 5c), but neither EGF receptor phosphorylation nor Ras activation were detected in EGF-treated p16INK4a-expressing cells. These results indicate that the expression of p16INK4a leads to the failure of EGF receptor-mediated Ras activation. This may be the mechanism responsible for the resisting phenotype of *Clk*/*Clk* mice to chemical carcinogen-induced tumorigenesis.

## Discussion

Consistent with our previous findings obtained using an oncogene-induced cellular transformation model^18^, the present results also revealed that the genetic ablation of the circadian gene *Clock* in mice resulted in resistance to chemically-induced tumorigenesis. During the development of primary cancers, chemical carcinogens induce cellular damage, apoptotic cell death, and inflammation. Consequently, cell growth and repair factors are released in the damaged and inflamed area for the restoration of tissues. The abnormal proliferation of epidermal cells occurred in the skin of DMBA-treated wild-type mice, and this appears to have been caused by EGF receptor-mediated Ras activation. Abnormal cell proliferation signals override the normal cell-cycle mechanism, resulting in tumor development in wild-type mice. On the other hand, epidermal cells in the skin of *Clk/Clk* mice may have difficulties tolerating excessive cell proliferation signals. The failure of epidermal cells in *Clk/Clk* cells to override the normal cell-cycle mechanism appeared to induce p16INK4a expression, thereby showing the SASP. Therefore, *Clk/Clk* mice exhibit the phenotype that resists chemically-induced tumorigenesis.

Tumorigenesis is known to proceed in three steps: initiation, promotion, and progression^54–56^. We previously reported a decrease in the expression of enzymes responsible for producing the active metabolite of diethylnitrosamine in the livers of *Clk/Clk* mice^57^. This led to the alleviation of DNA damage in the hepatocytes of *Clk/Clk* mice, even when they were exposed to chemical carcinogens. These facts indicate that a dysfunction in CLOCK influences the initiation step of chemically-induced tumorigenesis. However, no significant differences were observed in the DMBA-induced dermal expression of Cyp1a1 and 1b1 between wild-type and *Clk/Clk* mice. Furthermore, the amount of DMBA-DNA adducts in the skin of *Clk/Clk* mice was similar to that in wild-type mice. These findings suggest that the resistance of *Clk/Clk* mice to DMBA-induced tumorigenesis is not attributable to the initiation step.

Regarding the promotion step, a marked difference was observed in the activation of Ras between wild-type and *Clk/Clk* mice. The EGF receptor is a member of the ligand-activated receptor and tyrosine kinase family of transmembrane signaling proteins, which are fundamentally important regulators of cell growth and differentiation^58^. Previous studies reported that the EGF receptor is strongly expressed in epidermal cells, and its ligands are often up-regulated in the damaged or inflamed area of skin^59, 60^. Therefore, EGF receptor-mediated Ras activation in DMBA-treated mice appeared to have occurred in the epidermal layer. This notion is also supported by previous findings that DMBA-induced tumor development in the skin of mice is associated with the malignant transformation of epidermal cells^61, 62^. Although the DMBA treatment induced the expression of several types of ligands for the EGF receptor in wild-type and *Clk/Clk* mice, Ras activation was only observed in DMBA-treated wild-type mice. The genotype-dependent difference in Ras activation may also be reflected in the disparity in the degree of epidermal hyperplasia between wild-type and *Clk/Clk* mice.

The hallmark of cellular senescence is an inability to progress through the cell cycle. Senescent cells arrest growth irreversibly, but remain metabolically active^63^. Once arrested, cells fail to proliferate in response to adequate growth stimuli and often become resistant to cell death signals^64, 65^. The number of apoptotic cells in the skin of DMBA-treated *Clk/Clk* mice was smaller than that in wild-type mice; however, the treatment with DMBA induced the expression of p16INK4a only in *Clk/Clk* mice. We previously demonstrated that ATF4 is a potent repressor of the expression of p16INK4a^66^. Elevated levels of ATF4 in oncogene-introduced wild-type cells are sufficient to suppress the expression of p16INK4a and drive oncogenic transformation. On the other hand, CLOCK acts as a transcriptional activator of the *Atf4* gene^18^; thus, the expression levels of ATF4 in *Clk*/*Clk* cells remain low even when they receive oncogenic growth signals. Therefore, unresponsiveness of the *Atf4* gene to oncogenic stimuli allows the constitutive expression of p16INK4a in *Clk*/*Clk* cells, resulting in the induction of SASP reflecting the expression of *Ccl2*, *IL-6* and *Tnfα* ^18^. We also confirmed that ATF4 expression was not significantly induced in the skin of DMBA-treated *Clk/Clk* mice (Supplementary Fig. 1), which likely allow the expression of p16INK4a. Furthermore, transgenic expression of p16INK4a in human keratinocytes induced the SASP as well as prevented EGF receptor-mediated Ras activation. Therefore, the induction of p16INK4a expression appears to cause the failure of Ras activation in DMBA-treated *Clk/Clk* mice and their resisting phenotype to chemically carcinogen-induced primary cancer.

Since the basal mechanism of the circadian clock is well conserved in many mammalian species, CLOCK is assumed to function in almost the same manner in human cells. The present results suggest an uncovered role for CLOCK in the development of chemically-induced primary tumors and will contribute to new preventive strategies.

## Methods

### Treatment of animals

Female *Clk/Clk* mice with an ICR background and female wild-type mice of the same strain were housed in groups (from 3 to 6 per cage) in a light-controlled room (lights on from 7:00 to 19:00) at 24 ± 1 °C and humidity of 60 ± 10% with food and water *ad libitum*. Mice were used for experiments between the ages of seven and nine weeks. All protocols using mice were reviewed and approved by the Animal Care and Use Committee, Kyushu University. All methods were performed in accordance with the relevant guidelines and regulations.

### Cell culture and treatment

HaCaT cells were maintained in Dulbecco’s modified Eagle’s medium (Sigma-Aldrich, St. Louis, MO) supplemented with 10% fetal bovine serum (Thermo Fisher Scientific, Waltham, MA) and antibiotics at 37 °C in a humidified 5% CO_2_ atmosphere. Cells were infected with retroviral vectors expressing Flag-tagged p16INK4a (Addgene #24934). After growing cells to semi-confluence, serum was starved for 24 hours. EGF (Thermo Fisher Scientific) was added to media at a final concentration of 200 ng mL^−1^. One minute later, media were aspirated and cells were frozen by liquid nitrogen. Cells were used in the assessment of the active form of Ras.

### Chemically-induced skin tumor model

A chemically-induced tumor formation analysis was performed according to a previously described protocol with some modifications^25–27^. The dorsal skin of mice was shaved and, 1 day later, 100 μg of DMBA (Sigma-Aldrich) dissolved in 200 μL of acetone (Nacalai Tesque, Kyoto, Japan) was applied biweekly to the shaved area for 8 weeks. The diameters of tumors were measured using a caliper (Shinwa Rules Co., Ltd., Niigata, Japan) and the number of tumors with a diameter of more than 1 mm was counted once a week.

### Histological analysis

The dorsal skin of mice was shaved and 100 μg DMBA or vehicle (200 μL acetone) were applied biweekly for 2 weeks. Skin tissues were collected and fixed in 4% buffered formalin. Fixed skin tissues were transferred to 15% sucrose in PBS for 24 hours and then 30% sucrose in PBS for 24 hours. These samples were frozen in −80 °C and serially sectioned on a cryostat at a thickness of 14 μm. Sections were observed by microscopy after staining with hematoxylin (Muto Pure Chemicals Co., Ltd., Tokyo, Japan) and eosin (Muto Pure Chemicals Co., Ltd.).

### Western blotting

Skin tissues were homogenized with CelLytic^TM^ MT Cell Lysis Reagent (Sigma-Aldrich). Ten micrograms of the protein lysate was resolved by SDS-PAGE, transferred to a PVDF membrane, and probed with antibodies against PCNA (1:1,000; ab29, Abcam), Cyp1a1 (1:1,000; #871, Daiichi Pure Chemical Co., Ltd., Tokyo, Japan), Cyp1b1 (1:1,000; ab185954, Abcam, Cambridge, UK), Actin (1:1,000; sc1616-HRP, Santa Cruz Biotechnology, Santa Cruz, CA), Pan-Ras (1:1000; Component of STA-440, Cell Biolabs, Inc., San Diego, CA), EGF receptor (1:1000; #2232, Cell Signaling Technology, Danvers, MA), phosphorylated (Y1086) EGF receptor (1:1,000; ab32086, Abcam), MEK1 (1:1,000; ab32091, Abcam), phosphorylated (E342) MEK1 (1:1,000; ab396379, Abcam), Cleaved Caspase-3 (1:5000; #9661, Cell Signaling Technology), Flag-M2 (1:1000; F3165, Sigma-Aldrich),﻿﻿ and p16INK4a (1:500; sc1207, Santa Cruz Biotechnology). Specific antigen-antibody complexes were visualized using horseradish peroxidase-conjugated secondary antibodies and Chemi-Lumi One (Nacalai Tesque) or ImmunoStar reagent (Wako, Osaka, Japan).

### Measurement of DMBA-DNA adduct formation

The dorsal skin of mice was shaved and, 1 day later, 300 μg of DMBA dissolved in 200 μL of acetone was applied twice at 8-hour intervals. DNA was extracted from skin with the Genomic DNA Purification Kit (Promega, Madison, WI) 0 or 24 hours after the second application of DMBA. The fluorescence intensity of DMBA-DNA adducts was measured using a FlexStation3 (Molecular Devices, Sunnyvale, CA) with a 1/2 AreaPlate-96 (Black; Perkin Elmer, Waltham, MA). Emission data at 445 nm were obtained with excitation at 390 nm based on the spectrum previously reported^33^, and the intensity of fluorescence was normalized by DNA concentrations in the samples. DNA concentrations were measured using a nucleic acid analyzer (DW-K2800; Drawell International Technology Co., Ltd., PuDong, Shanghai, China).

### Measurement of the active form of Ras

The dorsal skin of mice was shaved and 100 μg DMBA or vehicle (200 μL acetone) was applied twice a week. Skin tissues were collected 2 weeks after the initiation of the DMBA treatment. One hundred micrograms of the EGF receptor antagonist AG1478 (Wako) was applied to the dorsal skin of mice 3 hours before sampling. The amount of the active form of Ras was measured using an ELISA Kit (STA-440; Cell Biolabs, Inc.). The values of absorbance at 450 nm were normalized by total protein concentrations in the samples. Protein concentrations were measured using the BCA assay (Thermo Fisher Scientific).

### Quantitative RT-PCR analysis

RNA was extracted from the skin of mice by RNAiso reagent (Takara Bio Inc., Osaka, Japan). In order to quantify the mRNA levels of each factor, cDNA was synthesized by reverse transcription using a ReverTra Ace quantitative real-time PCR kit (Toyobo, Osaka, Japan). Diluted cDNA samples were analyzed by PCR using a THUNDERBIRDSYBR qPCR Mix (Toyobo) and LightCycler® 96 System (Roche, Basel, Switzerland). Data were normalized using *18 s* mRNA as a control. PCR primer sequences are described in Supplementary Table 1.

### TUNEL staining

Skin sections were prepared using the same procedure described above. Apoptosis in skin sections was evaluated using a DeadEnd™ Fluorometric TUNEL System (Promega).

### Statistical and data analyses

The values presented are expressed as Means ± s.e.m.. The significance of differences in the total number of tumors between wild-type and *Clk/Clk* mice was tested by a two-way repeated measures ANOVA. The significance of differences among groups was analyzed by a one-way ANOVA followed by Tukey-Kramer’s post-hoc test. Equal variances were not formally tested. *P* < 0.05 was considered to be significant.

### Data availability

All data supporting the results of the present study are included in the article, either in the main figures or supplementary information files.

## Electronic supplementary material

Supplementary Data
