# Supplementary material for: Dysfunction of the circadian transcriptional factor CLOCK in mice resists chemical carcinogen-induced tumorigenesis
Source: Sci Rep. 2017 Aug 30;7:9995. doi: 10.1038/s41598-017-10599-1 (PMC5577256; doi:10.1038/s41598-017-10599-1)
Supplement: Supplementary file 1 — Supplementary Data [file 41598_2017_10599_MOESM1_ESM.pdf]

Supplementary information

Dysfunction of the circadian transcriptional factor CLOCK in mice resists chemical carcinogen-induced tumorigenesis

Ken-ichi Hashikawa[1], Chiharu Katamune[1], Naoki Kusunose[1], Naoya Matsunaga[1,2], Satoru Koyanagi[1,2], Shigehiro Ohdo[1]*

[1] Department of Pharmaceutics and [2] Department of Glocal Healthcare Science
Faculty of Pharmaceutical Sciences
Kyushu University
3-1-1 Maidashi Higashi-ku, Fukuoka 812-8582, Japan

* Correspondence should be addressed to S.O. (ohdo@phar.kyushu-u.ac.jp)

Supplementary Table 1　Primer sets for RT-PCR analysis.

Supplementary Figure 1　Protein levels of ATF4 in the skin of wild-type and *Clk*/*Clk* mice after the DMBA treatment.

Supplementary Figure 2　Unedited full blots of Figure 1d.

Supplementary Figure 3　Unedited full blots of Figure 2a.

Supplementary Figure 4　Unedited full blots of Figure 3b.

Supplementary Figure 5　Unedited full blots of Figure 4b.

Supplementary Figure 6　Unedited full blots of Figure 5a.

Supplementary Figure 7　Unedited full blots of Figure 5c.

Supplementary Figure 8　Unedited full blots of Supplementary Figure 1.

Supplementary Table 1   Primer sets for RT-PCR analysis

Gene	Primers
Mouse *Hb-egf*	Forward, 5'-TCGTCCGTCTGTCTTCTTGT-3'
	Reverse, 5'-GTAGCCTCTGAAGGTTCCTAT-3'
Mouse *Amphiregulin*	Forward, 5'-TTGGCATCGGCATCGTTAT-3'
	Reverse, 5'-CTTTTACCCTGCATTGTCCTCA-3'
Mouse *Tgf-α*	Forward, 5'-CTGGGTATCCTGTTAGCTGTGT-3'
	Reverse, 5'-GGGAATCTGGGCACTTGTTG-3'
Mouse *Egf*	Forward, 5'-ACTACTACAGGACTCGGAAGCA-3'
	Reverse, 5'-TGGGGTCTTGGTGTTTCTCT-3'
Mouse *IL-6*	Forward, 5'-CTGCAAGAGACTTCCATCCAG-3'
	Reverse, 5'-AGTGGTATAGACAGGTCTGTTGG-3'
Mouse *Tgf-β1*	Forward, 5'-TGACGTCACTGGAGTTGTACGG-3'
	Reverse, 5'-GGTTCATGTCATGGATGGTGC-3'
Mouse *Ccl2*	Forward, 5'-TTAAAAACCTGGATCGGAACCA-3'
	Reverse, 5'-GCATTAGCTTCAGATTTACGGG-3'
Mouse *Tnf-α*	Forward, 5'-CTGAACTTCGGGGTGATCGG-3'
	Reverse, 5'-GGCTTGTCACTCGAATTTTGAGA-3'
Human *IL-6*	Forward, 5'-ACTCACCTCTTCAGAACGAATTG-3'
	Reverse, 5'-CCATCTTTGGAAGGTTCAGGTTG -3'
Human *Tgf-β1*	Forward, 5'-GGCCAGATCCTGTCCAAGC-3'
	Reverse, 5'-GTGGGTTTCCACCATTAGCAC-3'
Human *Ccl2*	Forward, 5'-CAGCCAGATGCAATCAATGCC-3'
	Reverse, 5'-TGGAATCCTGAACCCACTTCT-3'
Human *Tnf-α*	Forward, 5'-GAGGCCAAGCCCTGGTATG-3'
	Reverse, 5'-CGGGCCGATTGATCTCAGC-3'
Mouse/Human *18s*	Forward, 5'-CGGCTACCACATCCAAGGAA-3'
	Reverse, 5'-GCTGGAATTACCGCGGCT-3'

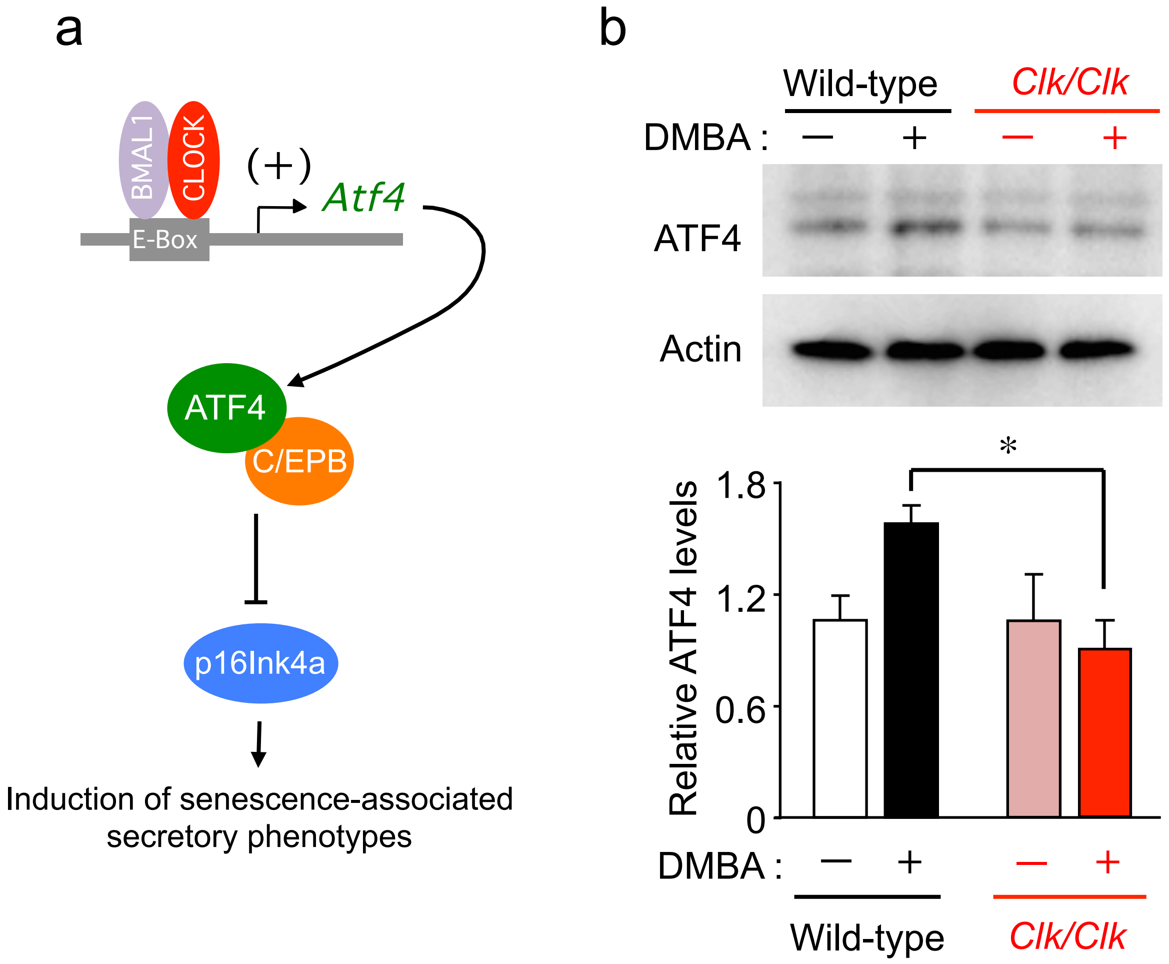

Supplementary Figure 1 Protein levels of ATF4 in the skin of wild-type and *Clk/Clk* mice after the DMBA treatment. (a) Schematic mechanism of CLOCK-mediated expression of ATF4 and its suppressive action on p16Ink4a-induced senescence-associated secretory phenotypes. The expression of ATF4 is activated by CLOCK/BMAL1 transcriptional activator complex. ATF4 forms with C/EPB and acts as a transcriptional repressor of p16Ink4a. (b) DMBA (100 μg) was applied biweekly to the back skin of mice for 2 weeks. Plus and minus indicate before and after DMBA treatment, respectively. Full-size images are presented in Supplementary Fig. 8. Values show the means ± s.e.m. (n = 3). *P < 0.05 significant difference between two groups ($F_{3,8}$ = 3.808, P = 0.058; ANOVA with Tukey-Kramer's post-hoc test).

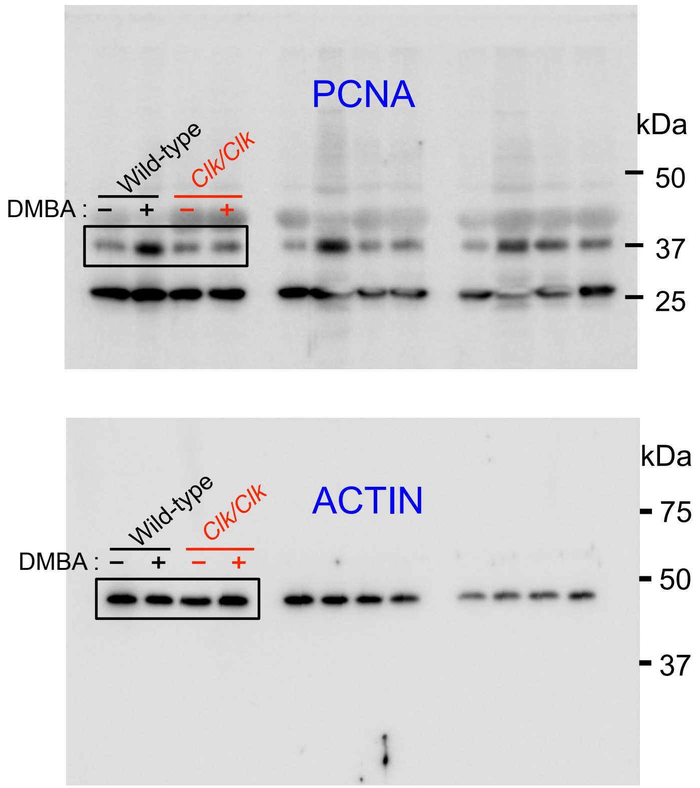

Supplementary Figure 2 Unedited full blots of Figure 1d

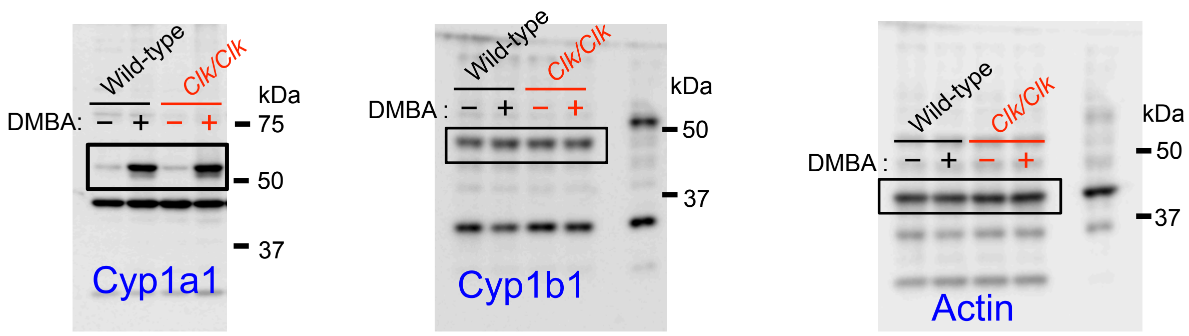

Supplementary Figure 3 Unedited full blots of Figure 2a

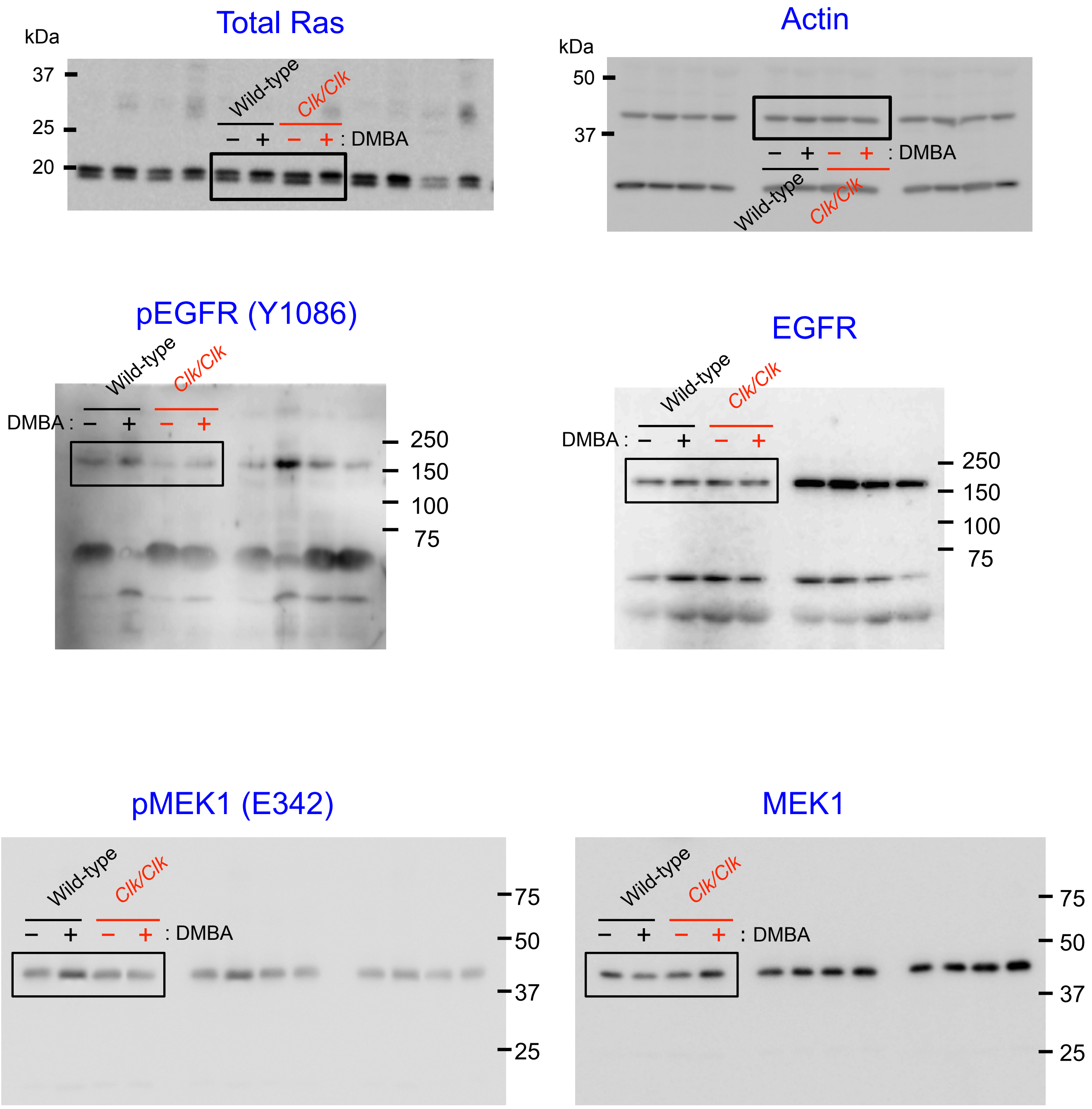

Supplementary Figure 4 Unedited full blots of Figure 3b

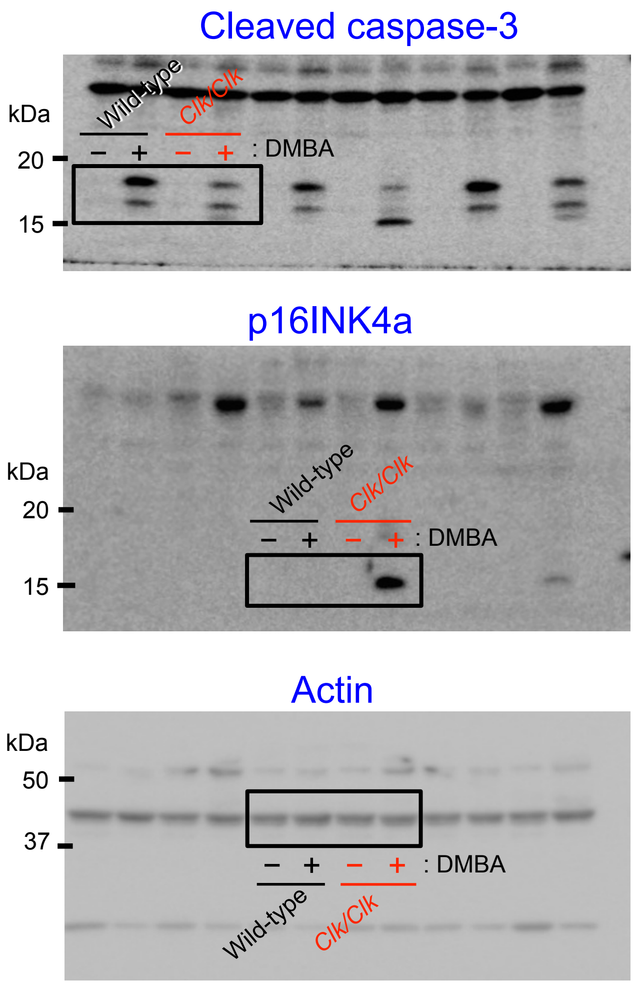

Supplementary Figure 5 Unedited full blots of Figure 4b

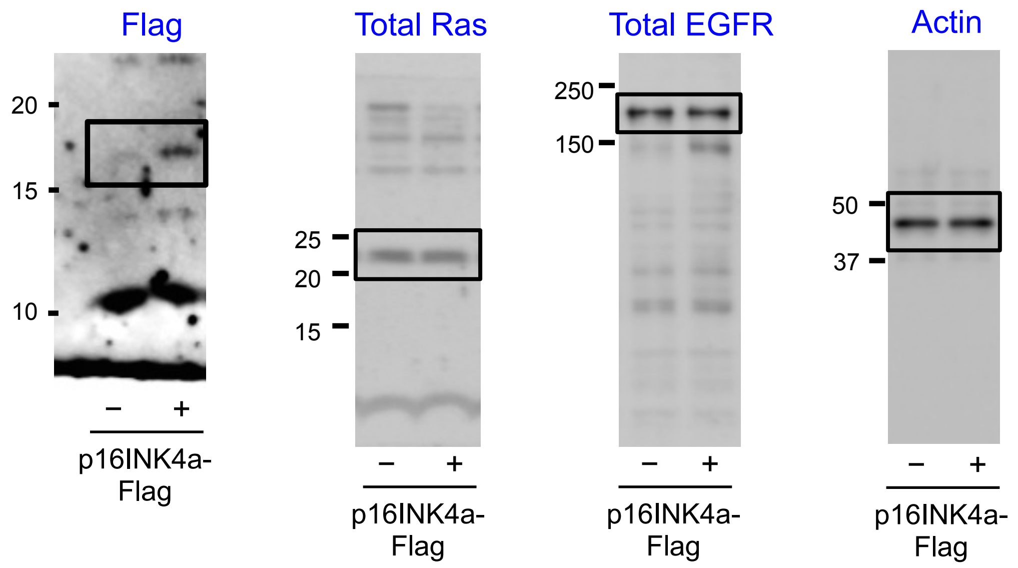

Supplementary Figure 6 Unedited full blots of Figure 5a

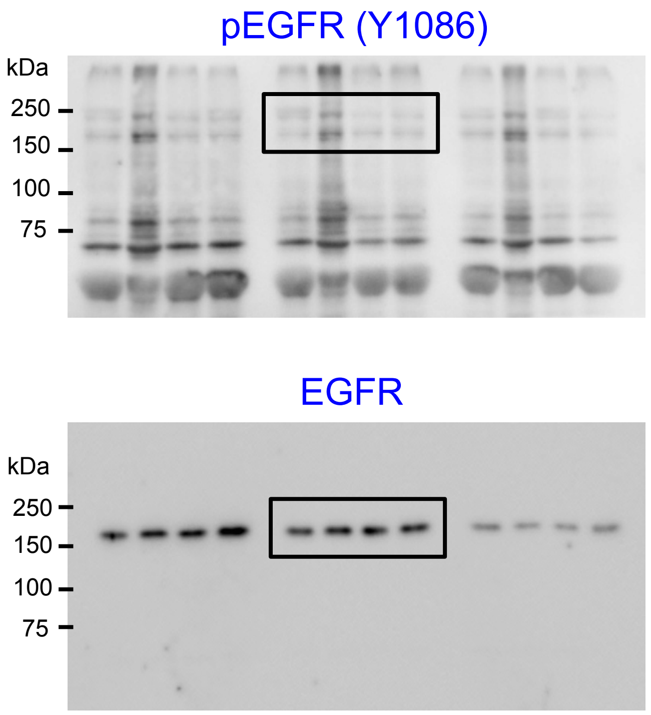

Supplementary Figure 7 Unedited full blots of Figure 5c

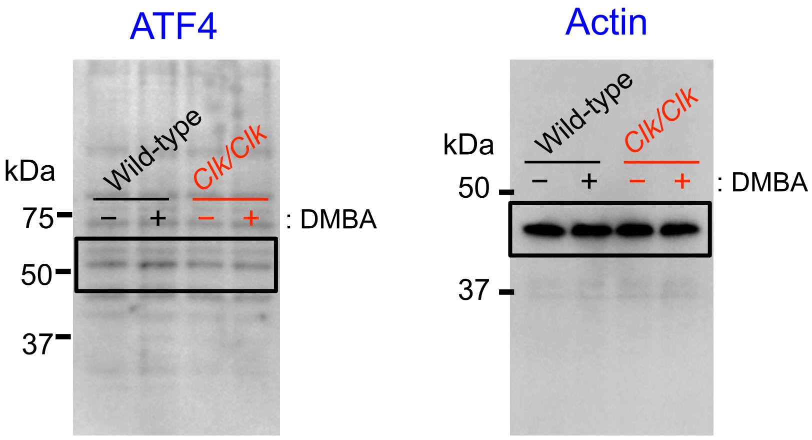

Supplementary Figure 8 Unedited full blots of Supplementary Figure 1
